# The effect of additional antimicrobial therapy on the outcomes of patients with idiopathic pulmonary fibrosis: a systematic review and meta-analysis

**DOI:** 10.1186/s12931-021-01839-0

**Published:** 2021-09-15

**Authors:** Ching-Yi Chen, Chao-Hsien Chen, Cheng-Yi Wang, Chih-Cheng Lai, Chien-Ming Chao, Yu-Feng Wei

**Affiliations:** 1 Division of Pulmonary Medicine, Department of Internal Medicine, E-Da Hospital, I-Shou University, Kaohsiung, Taiwan; 2 Division of Pulmonary, Department of Internal Medicine, MacKay Memorial Hospital, Taipei, Taiwan; 3 Department of Medicine, MacKay Medical College, New Taipei City, Taiwan; 4 Department of Internal Medicine, Cardinal Tien Hospital and School of Medicine, College of Medicine, Fu Jen Catholic University, New Taipei City, Taiwan; 5 Department of Internal Medicine, Kaohsiung Veterans General Hospital, Tainan Branch, Tainan, Taiwan; 6 Department of Intensive Care Medicine, Chi Mei Medical Center, Liouying, Tainan Taiwan; 7 Department of Internal Medicine, E-Da Cancer Hospital, Yan-Chao District, No. 21, Yida Road, Jiao-su Village, Kaohsiung, 824 Taiwan; 8 School of Medicine for International Students, College of Medicine, and Institute of Biotechnology and Chemical Engineering, I-Shou University, Kaohsiung, Taiwan

**Keywords:** Antibiotic, Antimicrobial agent, Co-trimoxazole, Doxycycline, Idiopathic pulmonary fibrosis, Outcome

## Abstract

**Background:**

The effect of additional antimicrobial agents on the clinical outcomes of patients with idiopathic pulmonary fibrosis (IPF) is unclear.

**Methods:**

We performed comprehensive searches of randomized control trials (RCTs) that compared the clinical efficacy of additional antimicrobial agents to those of placebo or usual care in the treatment of IPF patients. The primary outcome was all-cause mortality, and the secondary outcomes were changes in forced vital capacity (FVC), diffusing capacity of the lung for carbon monoxide (DLCO), and the risk of adverse events (AEs).

**Results:**

Four RCTs including a total of 1055 patients (528 receiving additional antibiotics and 527 receiving placebo or usual care) were included in this meta-analysis. Among the study group, 402 and 126 patients received co-trimoxazole and doxycycline, respectively. The all-cause mortality rates were 15.0% (79/528) and 14.0% (74/527) in the patients who did and did not receive additional antibiotics, respectively (odds ratio [OR] 1.07; 95% confidence interval [CI] 0.76 to 1.51; p = 0.71). No significant difference was observed in the changes in FVC (mean difference [MD], 0.01; 95% CI − 0.03 to 0.05; p = 0.56) and DLCO (MD, 0.05; 95% CI − 0.17 to 0.28; p = 0.65). Additional use of antimicrobial agents was also associated with an increased risk of AEs (OR 1.65; 95% CI 1.19 to 2.27; p = 0.002), especially gastrointestinal disorders (OR 1.54; 95% CI 1.10 to 2.15; p = 0.001).

**Conclusions:**

In patients with IPF, adding antimicrobial therapy to usual care did not improve mortality or lung function decline but increased gastrointestinal toxicity.

**Supplementary Information:**

The online version contains supplementary material available at 10.1186/s12931-021-01839-0.

## Background

Idiopathic pulmonary fibrosis (IPF) is a devastating progressive interstitial lung disease without an identifiable etiology [1]. The prevalence of IPF is increasing worldwide, particular in elderly populations [2–5]. Unlike other interstitial lung diseases, IPF is not likely to respond and maybe harmful to anti-inflammatory treatment with corticosteroids, and the prognosis is even worse than many cancers [6]. Therefore, IPF is associated with high morbidity and mortality, with a median survival of only 2–3 years from the time of diagnosis [6]. Large-scale randomized control trials (RCTs) [7, 8] have demonstrated that two anti-fibrotic agents, pirfenidone and nintedanib, can reduce the progression of IPF in lung function, exercise tolerance, and mortality. These two agents have obtained approval from the United States (US) Food and Drug Administration for the treatment of IPF and are widely used in the European Union (EU) and other countries worldwide. However, their usefulness may be limited by their high cost and difficult to tolerate toxicity [9–11].

Due to the limited treatment options for this fatal condition, an effective low-cost treatment is urgently needed to improve the clinical outcomes of patients with IPF. An earlier in vitro study showed that doxycycline could attenuate pulmonary fibrosis through the inhibition of growth factor and MMP production in alveolar epithelial cells [12]. Using a murine model, Kalemci et al. demonstrated that the administration of minocycline may be effective in methotrexate-induced lung fibrosis [13]. A pilot study of co-trimoxazole (trimethoprim-sulfamethoxazole) in 20 patients with progressive fibrotic lung disease demonstrated that additional treatment with co-trimoxazole resulted in a significant improvement in shuttle walking test and lung function in terms of forced vital capacity (FVC) [14]. In addition, in vitro studies have demonstrated that doxycycline and minocycline can improve pulmonary fibrosis by inhibiting growth factor and matrix metalloproteinase (MMP) production [12, 13]. Based on these promising findings, the effect of the additional use of antimicrobial agents such as doxycycline, co-trimoxazole, and macrolides on the outcomes of IPF patients have been assessed in further clinical studies [15–17]. Shulgina et al. reported a RCT of 181 IPF patients, and concluded that co-trimoxazole therapy could improve the quality of life and reduce mortality in those adhering to treatment [18]. In addition, a retrospective analysis of 209 IPF patients who received mechanical ventilation and high-dose corticosteroids showed that the concurrent use of co-trimoxazole (odds ratio [OR] 0.28, 95% confidence interval [Cl] 0.132–0.607; p = 0.001) and macrolides (OR 0.37, 95% Cl 0.155–0.867; p = 0.033) was significantly associated with reduced mortality [15]. Treating IPF with the addition of antimicrobial agents such as co-trimoxazole has also been shown to be cost-effective [19]. However, the findings of subsequent large RCTs [20, 21] have been inconsistent. Therefore, we conducted this systematic review and meta-analysis of RCTs to investigate the effect of additional antimicrobial agents on the clinical outcomes of patients with IPF.

## Methods

The study protocol was registered in the PROSPERO database with the ID number of CRD42021255619. This systematic review followed the Preferred Reporting Items for Systematic Reviews and Meta-Analyses (PRISMA) guidelines [22].

### Study search and selection

We performed a comprehensive search of the PubMed, Embase, Web of Science and Cochrane Library databases from their inception to May 20th 2021. The following search terms were used: idiopathic pulmonary fibrosis and antibiotics (including co-trimoxazole, tetracycline, chlortetracycline, oxytetracycline, demeclocycline, lymecycline, meclocycline, methacycline, metacycline, minocycline, rolitetracycline, doxycycline, tigecycline, eravacycline, sarecycline, omadacycline, azithromycin, clarithromycin, erythromycin, fidaxomicin). The clinical trials registries of ClinicalTrials.gov and WHO International Clinical Trials Registry Platform for relevant articles were also searched. The detailed search strategy is described in Additional file 1: Table S1. Only RCTs that compared the clinical efficacy and safety of additional antimicrobial agents to those of placebo or usual care in the treatment of patients with IPF were included. The reference lists of relevant articles were also searched manually for additional eligible articles. No language limitations were applied.

### Study selection and data extraction

Three investigators (CHC, CYC, CCL) independently screened and reviewed each study. Studies were included if they met the following criteria: (1) adult patients with IPF, (2) intervention of additional antimicrobial agents, (3) comparisons with placebo or usual care, (4) RCTs, and (5) efficacy outcome with or without safety. We excluded in vitro activity research, animal studies, and pharmacokinetic–pharmacodynamic assessments. If there were any disagreements, fourth and fifth investigators (CYW and FYW) were consulted. For each included study, we extracted the following data: year of publication, study design, antimicrobial regimens, clinical outcomes, and risk of adverse events (AEs).

### Outcome measurements

The primary outcome was all-cause mortality, and the secondary outcomes were changes in FVC, diffusing capacity of the lung for carbon monoxide (DLCO), and the risk of AEs.

### Quality assessment and data analysis

The Cochrane risk-of-bias tool was used to assess the quality and associated risks of bias of the included RCTs [23]. Two reviewers independently reviewed all of the included studies on the following items: randomization sequence generation, allocation concealment, blinding of participants and personnel, blinding of outcome assessment, incomplete outcome data, selective reporting, and inclusion of intention-to-treat analyses, and rated them as “low risk,” “high risk,” or “unclear risk”. If there was any disagreement, a third reviewer was consulted and a decision was reached by consensus.

Statistical analyses were performed using Review Manager (version 5.3; Nordic Cochrane Centre, Copenhagen, Denmark). Heterogeneity among the included studies was assessed using Cochran’s Q test and *I*^2^ statistic, and a p-value < 0.05 was considered to be statistically significant. When *I*^2^ < 50%, a fixed effects model was used, otherwise a random effects model was used. Pooled odds ratios (ORs), mean differences (MDs) and 95% confidence intervals (CIs) were calculated for outcome analyses. Sensitivity analyses were performed to assess the contribution of each study by excluding one individual study and recalculating the pooled hazard ratio estimates for the remaining studies (leave-one-out meta-analysis).

## Results

### Study selection

**Fig. 1:**
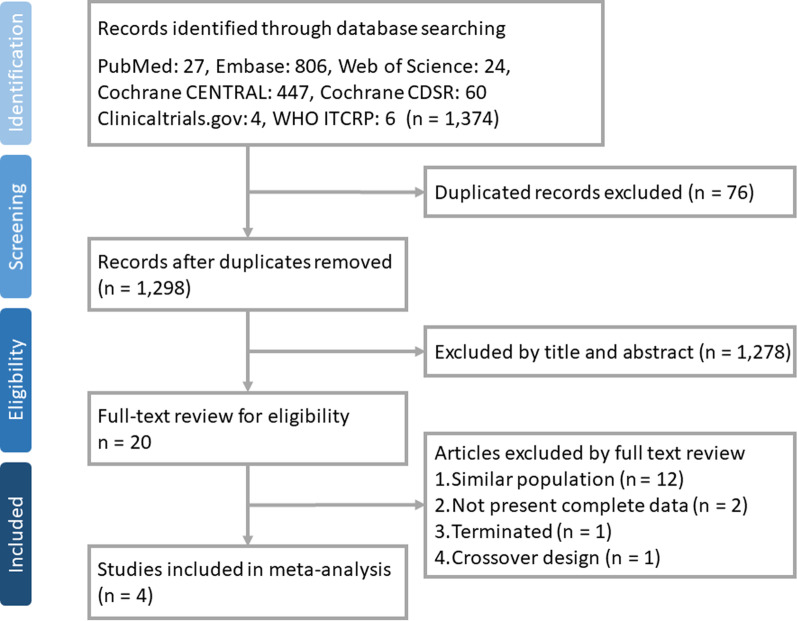
Flow diagram showing the identification of eligible trials and participating trials

The search results yielded a total of 1374 studies from the online databases including PubMed (n = 27), Web of Science Core Collection (n = 24), Embase (n = 806), Cochrane Library (n = 507), clinicaltrials.gov (n = 4), and WHO International Clinical Trials Registry Platform (n = 6) (e-Table 1). Seventy-six studies were excluded as duplicates, 1278 studies were found to be irrelevant after the title and abstract were screened, and 16 studies were excluded for having the same population, terminated (NCT01777737), no complete data available (NCT00203697 and EUCTR2014-004058-32) and using a crossover design (NCT02173145) after the full text had been screened. Finally, four RCTs [14, 18, 20, 21] were included in this meta-analysis (Fig. 1).

### Study characteristics

The characteristics of the four included RCTs are summarized in Table 1. Three studies [14, 18, 20] were double-blind and placebo-controlled design. Three RCTs [14, 18, 20] were conducted in the EU and one [21] was conducted in the US. Co-trimoxazole was the only studied antibiotic in three RCTs [14, 18, 20], and one RCT [21] used co-trimoxazole or doxycycline as the experimental drug. The duration of additional antibiotic treatment varied among the four RCTs [14, 18, 20, 21]. Overall, a total of 1055 patients, including 528 who received additional antibiotics and 527 who received placebo or usual care were included in this meta-analysis. Among the study group, 402 and 126 patients were randomly assigned to receive co-trimoxazole or doxycycline, respectively.

**Table 1:** Characteristics of the included studies

Study	Design	Study site	Study period	Study subjects	Study drug	Control	No. in study group	No. in control group	Primary outcome
Varney et al. 2008 [14]	Double-blind randomized placebo-controlled pilot study	Single center in the UK	NA	Patients < 85 years old with progressive fibrotic lung disease	co-trimoxazole for 3 months	Placebo	10	10	Exercise capacity
Shulgina et al. 2013 [18]	Randomized placebo-controlled double-blind parallel-group	28 sites in the UK	2008–2009	Patients aged > 40 years, with a diagnosis of fibrotic idiopathic interstitial pneumonia and a MRC dyspnea score of ≥ 2	co-trimoxazole for 12 months	Placebo	95	86	Forced vital capacity
Wilson et al. 2020 [20]	Double-blind placebo-controlled, parallel randomized trial	43 sites in the UK	2015–2019	IPF diagnosed according to contemporaneous international guidelines and an mMRC dyspnea scale score > 1	co-trimoxazole for between 12 and 42 months	Placebo	170	172	Time to all-cause death, lung transplant, or first nonelective hospital admission
Martinez et al. 2021 [21]	Pragmatic, randomized, unblinded clinical trial	35 sites in the US	2017–2019	Aged ≥ 40 years and diagnosed with IPF by the enrolling investigator	co-trimoxazole or doxycycline + usual care	Usual care	254	259	Time to first nonelective respiratory hospitalization or all-cause mortality

*IPF*, idiopathic pulmonary fibrosis; *mMRC* modified Medical Research Council; *NA* not applicable

### Quality assessment

**Fig. 2:**
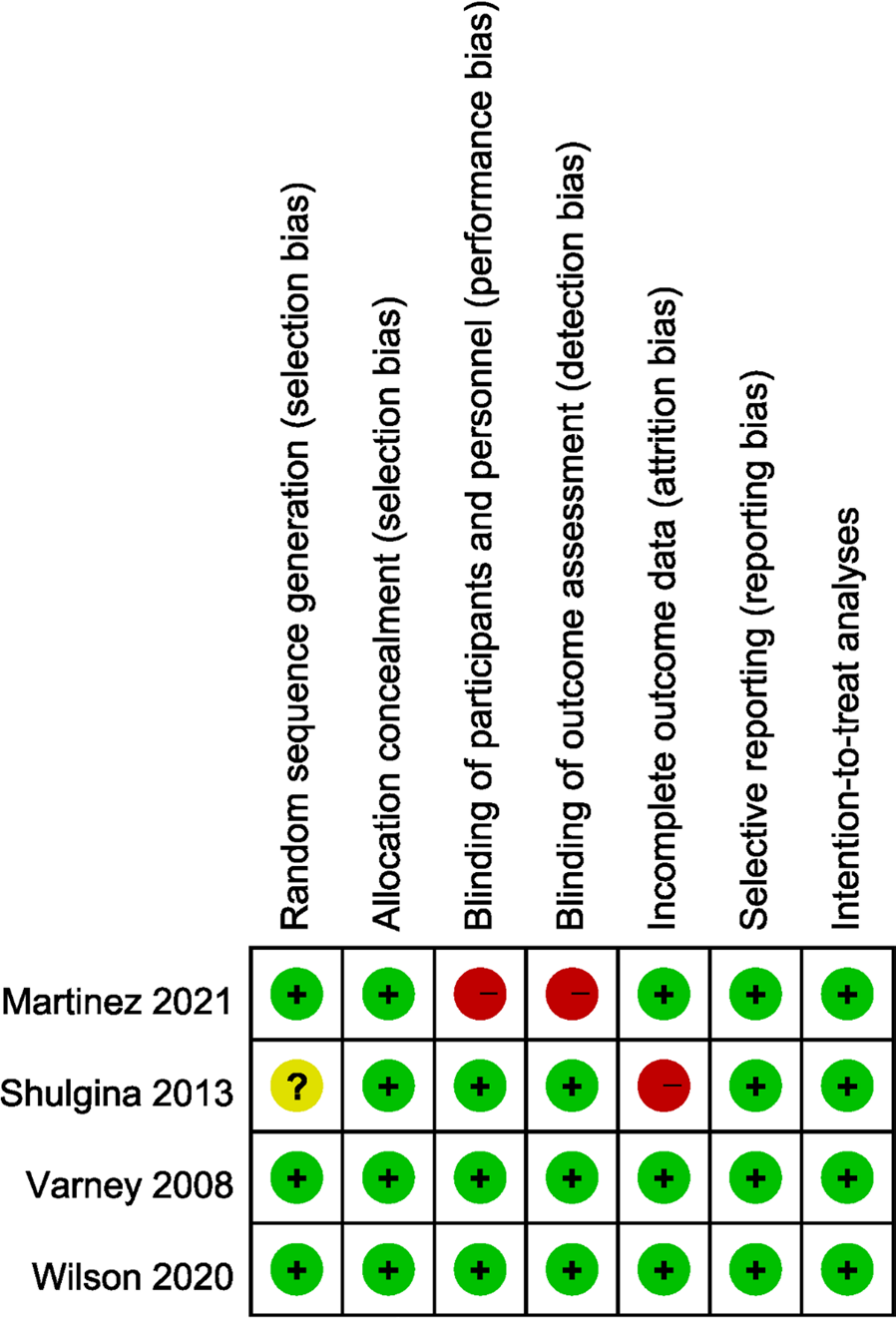
Summary of risks of bias in each domain for each included study

There were risks of performance and detection bias due to the open labelled design in one study [21]. Another study did not describe the details of random sequence generation, and it only reported the outcome data of 123 of 181 randomized patients [18]. However, the author did perform imputation sensitivity analysis, which revealed that the results were robust even with the missing data. A summary of the risk of bias for the included studies [14, 18, 20, 21] is depicted in Fig. 2.

### Mortality

**Fig. 3:**
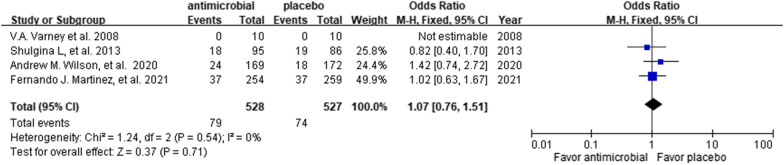
Forest plot of the comparison of all-cause mortality between the study and control groups

Overall, the all-cause mortality rates were 15.0% (79/528) and 14.0% (74/527) among in the patients who did (study group) and did not (control group) receive additional antibiotics, respectively. As shown in Fig. 3, no significant difference was observed in mortality between the study and control groups (OR 1.07; 95% CI 0.76 to 1.51; p = 0.71; *I*^2^ = 0%). The leave-one-out sensitivity analysis revealed that the magnitude of association between additional antibiotics with mortality was not influenced by individual studies.

### Secondary outcomes

**Fig. 4:**
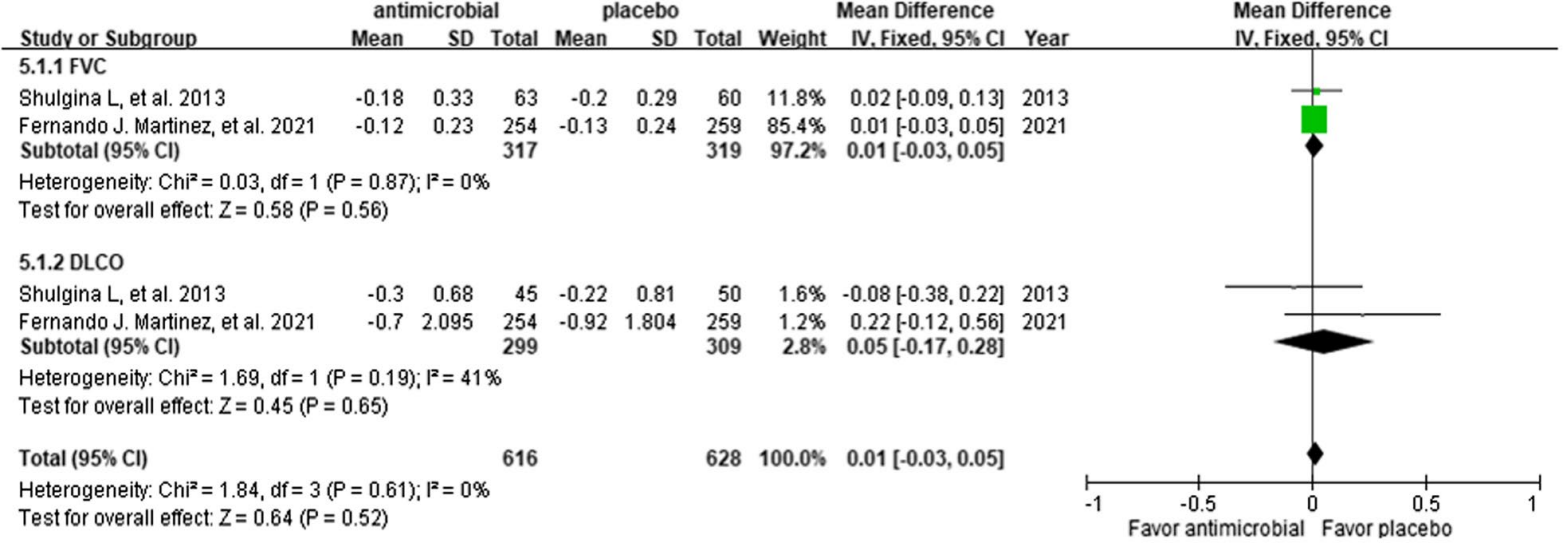
Forest plot of the comparisons of FVC and DLCO between the study and control groups

Compared to the control group, the use of additional antimicrobial agents was associated with mild improvements in FVC (MD 0.01; 95% CI − 0.03 to 0.05; p = 0.56; *I*^2^ = 0%) and DLCO (MD 0.05; 95% CI − 0.17 to 0.28; p = 0.65; *I*^2^ = 41%); however, these differences did not reach statistical significance (Fig. 4).

### Adverse events

**Fig. 5:**
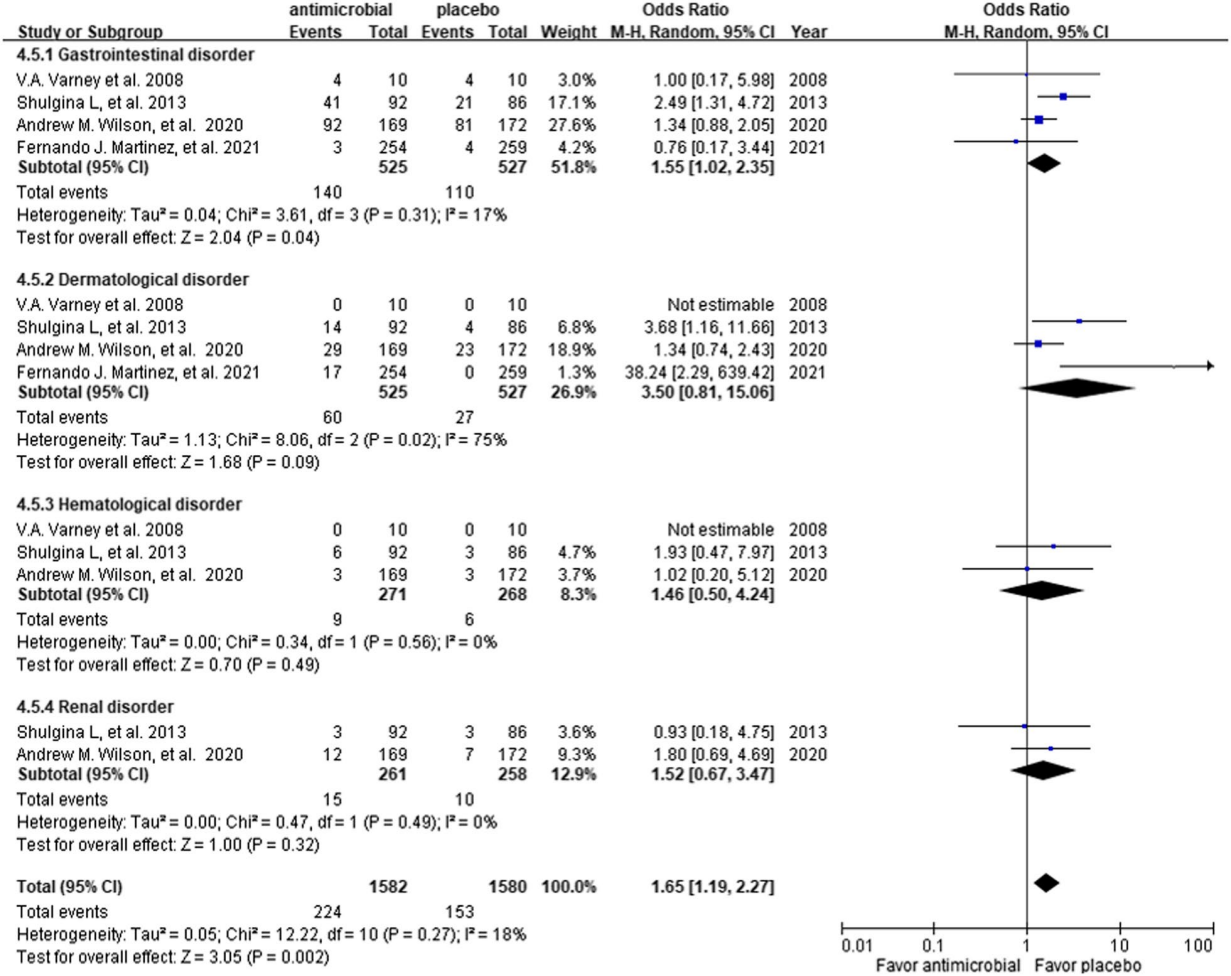
Forest plot of adverse events between the study and control groups

Compared to the control group, the use of additional antimicrobial agents was associated with an increased risk of AEs (OR 1.65; 95% CI 1.19–2.27; p = 0.002; *I*^2^ = 18%) (Fig. 5). Among these AEs, there was a significant risk of gastrointestinal disorders (OR 1.54; 95% CI 1.10–2.15; p = 0.001; *I*^2^ = 17%) and a trend of an increased risk of dermatological disorders (OR 3.50; 95% CI 0.81–15.06; p = 0.09; *I*^2^ = 75%). There were no significant differences in hematological (OR 1.47; 95% CI 0.51–4.22; p = 0.47; *I*^2^ = 0%) and renal (OR 1.53; 95% CI 0.67–3.46; p = 0.31; *I*^2^ = 0%) disorders.

## Discussion

In this meta-analysis, we reviewed four RCTs [14, 18, 20, 21] which compared the use of additional antimicrobial agents with placebo or usual care in terms of efficacy and safety for the treatment of adult patients with IPF. Our findings showed that antimicrobial agents (co-trimoxazole or doxycycline) did not provide additional benefits for patients with IPF in terms of mortality and FVC. In contrast, these agents were significantly associated with a higher risk of AEs, especially gastrointestinal toxicity. Based on these findings, the additional use of antimicrobial therapy for patients with IPF is not recommended.

Anti-fibrotic agents including pirfenidone and nintedanib have been approved and are widely used in several countries for the treatment of IPF. However, these two agents may not be available or affordable in certain countries. Immunomodulatory agents including azathioprine, colchicine, cyclophosphamide and interferon-gamma 1b have been investigated for the treatment of IPF, however, they have failed to show treatment benefits [24]. A previous systematic review and meta-analysis investigated pirfenidone, nintedanib and anti-oxidative therapy with N-acetylcysteine (NAC) for the treatment of IPF, showed that NAC was not significantly effective in reducing FVC decline over 12 months and that NAC provided a signal for increased adverse events [25]. A recent meta-analysis also indicated that add-on NAC to pirfenidone did not affect outcomes compared to pirfenidone alone [26].

In patients with IPF, Mishra et al. showed that doxycycline therapy reduced the levels of MMP-9, MMP-3, tissue inhibitor of metalloproteinase-1 and vascular endothelial growth factor in bronchial alveolar lavage fluid to near control values [16]. Previous studies have also demonstrated that the lung microbiome and bacterial burden can influence disease progression and outcomes in patients with IPF [27, 28].

However, in the current meta-analysis, we found that the additional use of antimicrobial agents did not provide clinical benefits in patients with IPF. The reason for our negative findings could be multifactorial. First, our findings were based on the analysis of intention-to-treat populations, not per-protocol populations, and adherence to the study medications was poor in the included RCTs. In Shulgina’s trial [18], the adherence rate to the study medication (co-trimoxazole) was only 66.3% (63/95). In the EME-TIPAC trial of 169 patients randomized to receive co-trimoxazole [20], 67 (39.6%) patients discontinued the medication and 26 (15.4%) reduced the dose. In the CleanUP-IPF trial [21], adherence to the study medication at 12 months was only 47.2% among 163 patients, and only 49.5% of the study patients were followed up for more than 12 months. In addition, the findings of per-protocol analysis in Shulgina’s study showed that co-trimoxazole was associated with a significant reduction in all-cause mortality (hazard ratio 0.21; 95% CI 0.06 to 0.78; p = 0.02) even though co-trimoxazole did not show a survival benefit based on the analysis of the intention-to-treat populations [18]. Therefore, these findings raise the question of whether the insignificant effect of additional antimicrobial therapy could be due to poor adherence, and further studies with better adherence are warranted to solve this issue. Second, there was heterogeneity among the included RCTs, including the characteristics of each study population. The diagnostic criteria of IPF, severity of baseline lung function, underlying comorbidities, and treatment duration varied among these studies. Furthermore, anti-fibrotic agents were not available in two earlier studies [14, 18], and more than 70% of the IPF patients received anti-fibrotic agents in the two most recent studies [20, 21]. Whether anti-fibrotic agents can influence the effect of anti-microbial agents is unknown. In addition, none of the included studies measured the number or type of lung microbes or the direct effect of antimicrobial agents on these microbiota, and therefore we cannot exclude the potential effect of antibiotics in selected patients with IPF and dysbiosis.

In terms of safety analysis, we found that the use of additional antimicrobial agents was significantly associated with a higher risk of AEs, especially gastrointestinal toxicity including diarrhea and vomiting, and a trend of dermatological side effects such as skin rash. These AEs were expected to be higher in the experimental group, however most were not fatal or serious. For other AEs, there were increased risks of hematological and renal disorders such as hyperkalemia and impaired renal function, although these differences did not reach statistical significance. However, the included patients in the trials were highly selected, and additional side effects associated with antimicrobial agents still need to be cautiously monitored in a clinical setting.

This meta-analysis had several limitations. First, the numbers of included studies and patients were relatively small, and adherence to the study medication was poor as mentioned above. Second, the antimicrobial agents were limited to co-trimoxazole or doxycycline, and the potential anti-bacterial benefits of co-trimoxazole may have been reduced due to widespread bacterial resistance. Other antibiotics such as macrolides and fluoroquinolones were not investigated in large studies. Third, we did not assess the effect of additional antimicrobial agents on other outcomes such as exercise tolerance with the six-minute walk test, risk of exacerbations or hospitalization, and quality of life because of a lack of data or differences in the measuring tools in different studies. However, there were no statistically significant differences in respiratory hospitalization or patient-reported outcomes including symptom scores and quality of life in two included studies [20, 21]. The primary outcome in this meta-analysis demonstrated no significant difference in mortality in the patients who received additional antimicrobial agents, and this finding remained unchanged in leave-one-out sensitivity analysis, which could strengthen the results of this meta-analysis.

## Conclusion

In conclusion, among patients with IPF, this meta-analysis indicated that adding antimicrobial therapy did not improve mortality or decline in lung function compared with placebo or usual care. In addition, these agents were significantly associated with a higher risk of AEs, especially gastrointestinal toxicity. These findings do not support the use of additional antimicrobial agents for improving the outcomes of patients with IPF.

## Supplementary Information

**Additional file 1: **PRISMA-2009-checklist_Wei.

## Data Availability

The datasets used and analyzed in the current study are available from the corresponding author on reasonable request.

## Abbreviations

AE: Adverse event
CI: Confidence interval
DLCO: Diffusing capacity of the lung for carbon monoxide
FVC: Forced vital capacity
IPF: Idiopathic pulmonary fibrosis
MD: Mean difference
MMP: Matrix metalloproteinase
OR: Odds ratio
RCT: Randomized control trial
